# Sex-Specific Associations of *MDM2* and *MDM4* Variants with Risk of Multiple Primary Melanomas and Melanoma Survival in Non-Hispanic Whites

**DOI:** 10.3390/cancers15102707

**Published:** 2023-05-11

**Authors:** Sarah V. Ward, Isidora Autuori, Li Luo, Emily LaPilla, Sarah Yoo, Ajay Sharma, Klaus J. Busam, David W. Olilla, Terence Dwyer, Hoda Anton-Culver, Roberto Zanetti, Lidia Sacchetto, Anne E. Cust, Richard P. Gallagher, Peter A. Kanetsky, Stefano Rosso, Colin B. Begg, Marianne Berwick, Nancy E. Thomas, Irene Orlow

**Affiliations:** 1 Memorial Sloan Kettering Cancer Center, New York, NY 10065, USA; 2 School of Population and Global Health, The University of Western Australia, Perth, WA 6009, Australia; 3 Department of Internal Medicine, The University of New Mexico Comprehensive Cancer Center, Albuquerque, NM 87106, USA; 4 Lineberger Comprehensive Cancer Center, University of North Carolina, Chapel Hill, NC 27514, USA; 5 Clinical Sciences Theme, Heart Group, Murdoch Children’s Research Institute, Melbourne, VIC 3052, Australia; 6 Nuffield Department of Women’s & Reproductive Health, University of Oxford, Oxford OX3 9DU, UK; 7 Faculty of Medicine, Dentistry and Health Sciences, University of Melbourne, Carlton, VIC 3010, Australia; 8 Oxford Martin School, University of Oxford, Oxford OX1 3BD, UK; 9 Menzies Institute for Medical Research, University of Tasmania, Hobart, TAS 7000, Australia; 10 Department of Medicine, University of California, Irvine, CA 92617, USA; 11 Piedmont Cancer Registry, Centre for Epidemiology and Prevention in Oncology in Piedmont, 10126 Turin, Italy; 12 The Daffodil Centre, The University of Sydney, A Joint Venture with Cancer Council NSW, Sydney, NSW 2006, Australia; 13 Melanoma Institute Australia, The University of Sydney, Wollstonecraft, NSW 2065, Australia; 14 BC Cancer Research Institute, Vancouver, BC V5Z 1L3, Canada; 15 Department of Dermatology and Skin Science, University of British Columbia, Vancouver, BC V5Z 4E8, Canada; 16 Department of Cancer Epidemiology, H. Lee Moffitt Cancer Center & Research Institute, Tampa, FL 33612, USA; 17 Department of Dermatology, University of North Carolina, Chapel Hill, NC 27514, USA

**Keywords:** *MDM2*, *MDM4*, melanoma, gene, polymorphism, risk, survival, sex, functional, estrogen-receptor

## Abstract

**Simple Summary:**

Melanoma is the most severe type of skin cancer, and the risk of developing melanoma and of dying of melanoma varies between women and men. This study investigated whether widely studied genetic single nucleotide polymorphisms (SNPs) in two oncogenes known to promote cell proliferation explain these sex differences by testing such variants in a large cohort of 3663 melanoma patients. Formal analyses demonstrated that females, but not males, who carried the variant *MDM4*-rs4245739*C were more likely to develop a new primary melanoma, and those with the variant *MDM2*-rs2279744*G were less likely to succumb to the disease. We also identified a number of additional variants, often co-inherited with the tested SNPs, which modify how these and other genes work locally in the skin, as well as in distal organs where melanoma tends to spread or invade during the progression of the disease.

**Abstract:**

*MDM2*-SNP309 (rs2279744), a common genetic modifier of cancer incidence in Li-Fraumeni syndrome, modifies risk, age of onset, or prognosis in a variety of cancers. Melanoma incidence and outcomes vary by sex, and although SNP309 exerts an effect on the estrogen receptor, no consensus exists on its effect on melanoma. MDM2 and MDM4 restrain p53-mediated tumor suppression, independently or together. We investigated SNP309, an *a priori MDM4*-rs4245739, and two coinherited variants, in a population-based cohort of 3663 primary incident melanomas. Per-allele and per-haplotype (MDM2_SNP309-SNP285; MDM4_rs4245739-rs1563828) odds ratios (OR) for multiple-melanoma were estimated with logistic regression models. Hazard ratios (HR) for melanoma death were estimated with Cox proportional hazards models. In analyses adjusted for covariates, females carrying *MDM4*-rs4245739*C were more likely to develop multiple melanomas (OR_per-allele_ = 1.25, 95% CI 1.03–1.51, and P_trend_ = 0.03), while *MDM2*-rs2279744*G was inversely associated with melanoma-death (HR_per-allele_ = 0.63, 95% CI 0.42–0.95, and P_trend_ = 0.03). We identified 16 coinherited expression quantitative loci that control the expression of *MDM2*, *MDM4*, and other genes in the skin, brain, and lungs. Our results suggest that *MDM4/MDM2* variants are associated with the development of subsequent primaries and with the death of melanoma in a sex-dependent manner. Further investigations of the complex *MDM2*/*MDM4* motif, and its contribution to the tumor microenvironment and observed associations, are warranted.

## 1. Introduction

Cutaneous melanoma is the most aggressive type of skin cancer. Its incidence, predominantly in fair-skinned populations, differs consistently between men and women, being more prevalent in women younger than 45 years and in men older than 69 years [1]. The total number of melanoma deaths is estimated at 7650 in the United States for 2022 [1], and although women are diagnosed at a younger age, they have better survival than men [1,2]. In addition to the stage at diagnosis, anatomic site, and exposures (including hormones), other factors seem responsible for the observed sex differences. A tumor–host interaction is likely involved; however, host determinants potentially contributing to the observed sex-specific differences have not been systematically investigated or in-depth, and the existing evidence is limited and inconsistent across studies [3,4,5,6,7,8,9,10,11,12,13].

A common variant in the human homolog of the mouse double minute 2 (MDM2) gene—commonly referred to as SNP309—overlaps with a specific protein 1 (Sp1) transcription factor binding site (TFBS), with the G allele resulting in elevated *MDM2* mRNA and Mdm2 protein levels [14,15], thereby attenuating the p53- and p73-mediated cell cycle arrest and apoptosis. We and others reported on the associations between SNP309 and risk for melanomas [10,16] as well as age at diagnosis [10,13]; however, after adjusting for covariates, in our hospital-based cohort, we found that women carrying the SNP309*G allele were at lower risk of developing melanoma at a younger age [13]. We also observed a decreased risk of dying of melanoma in patients carrying the G-allele; however, the effect was not statistically significant [13]. Other studies found no effect on either risk, age at diagnosis, or progression [9,11,12]. In addition to differences in sample size, stage of the disease, and adjustment (or lack thereof) for relevant covariates, we concluded that a nearby functional *MDM2* variant might have been responsible for the inconsistencies across studies. Indeed, the nearby *MDM2* promoter SNP285 is in linkage disequilibrium (LD) with SNP309, overlaps with another Sp1 TFBS, and the SNP285*C allele reduces the binding affinity of Sp1, decreasing the levels of Mdm2 [17].

One other oncoprotein, Mdm4 (also known as Mdmx), inhibits the p53 pathway by direct binding to and inhibiting p53’s transcriptional activity, enhancement of Mdm2, and inhibiting Mdm2’s self-ubiquitination [18,19]. The C allele of *MDM4*-rs4245739 creates an illegitimate binding site for the microRNA miR-191 [20,21]. This, in turn, inhibits *MDM4*’s translation, resulting in decreased expression of *MDM4* in carriers of C alleles. Conversely, the *MDM4*-rs4245739*A allele has been linked to increased *MDM4* expression and poorer outcomes in ovarian cancer [20].

Considering the important potentiating inhibitory role of Mdm2 and of Mdm4 on cell cycle regulation and shed clarity on the inconsistent and limited body of melanoma literature, here we aimed to investigate the overall and sex-specific effect of four *a priori* selected candidate variants and their haplotypes, on the risk of developing subsequent primary melanomas and on disease-specific survival in a large population-based melanoma study.

We also searched for additional candidate functional *MDM2/MDM4* variants in high LD with our tested single nucleotide polymorphisms (SNPs) that contribute to the genetic environment and create more or less favorable conditions in skin and/or distal tissues prone to melanoma metastases.

## 2. Materials and Methods

### 2.1. Study Population

Participants of the Genes, Environment, and Melanoma (GEM) Study were 3663 patients of European descent diagnosed with an incident single primary melanoma (SPM) (n = 2458) or multiple primary melanomas (MPM) (n = 1205) between 1998 and 2003. The GEM study has been previously described in detail [22]. Briefly, GEM is an international study that recruited patients from eight population-based cancer registries and one hospital center in Australia, Canada, Italy, and the United States of America. The study employs a design in which patients with newly diagnosed primary and invasive SPM were recruited as ‘controls’, and patients with newly diagnosed second or higher-order MPM were recruited as ‘cases’. The study design was validated by calculating odds ratios for well-known risk factors for melanoma and by comparing the obtained risk estimates in GEM to those attained in traditional case-control studies. We observed very similar risk estimates in GEM, as reported by Begg et al. (2006), for all skin characteristics with the exception of eye color and with ORs ranging, on average, 20% to 40% lower than OR estimates reported in other studies with a traditional design [23]. Genetic risks in GEM have also reflected population-based estimates obtained in other traditional case-control studies [22].

### 2.2. Biospecimens and Genotyping

Germline DNA was previously isolated from buccal brushes [23] and banked. The variants *MDM2* rs2279744, known as SNP309 for its position, and *MDM4* rs4245739, were selected primarily based on their disease associations and reports on their predicted and observed effect on expression, as summarized in the background [17,20,21]. *MDM2* rs117039649 (also known as SNP285) was included due to its high genetic linkage to SNP309 and known functional relevance, and *MDM4* rs1563828 was also included due to its high LD with rs4245739. These four SNPs were typed in 3663 samples, corresponding to 2458 SPM cases and 1205 MPM using the MassARRAY iPLEX chemistry and platform (Sequenom, San Diego, CA, USA). Assay conditions can be provided upon request. Standard quality control and quality assurance procedures were followed and described elsewhere [24]. Briefly, assays were considered successful based on the reproducibility of data (intra and inter-batch replicas agreement), clustering, lack of contamination, and specificity (confirmed through Sanger sequencing during assay development). The observed allelic frequencies were similar to those reported in the White non-Hispanic population (Supplementary Table S1). After excluding inconclusive signals and failures, genetic data were available for 3616 and 3457 melanoma cases–for MDM2 rs117039649/SNP285 and rs2279744/SNP309 respectively, and for 3575 and 3574 cases–for MDM4 rs1563828 and rs4245739. Data were reported as genotypes and haplotypes. For each gene, haplotype blocks in strong LD were estimated using Haploview 4.2 [25], and haplotype frequencies were estimated using PHASE v2.1 [26,27].

### 2.3. Statistical Analyses

Means, frequencies, and proportions were used to summarize the distributions of key participant characteristics from all GEM participants. Logistic regression models were used to estimate Odds Ratios (ORs) and 95% Confidence Intervals (CIs) for the effect of each individual SNP and haplotype on the risk of developing multiple melanomas by comparing participants with MPM (cases) on participants with SPM cases (controls). Analyses were conducted in all participants and in participants stratified by sex. An additive model of inheritance was assumed for the SNPs, and analyses were adjusted for age at diagnosis, sex, age-by-sex interaction, and study center. Interaction analyses conducted in the overall sample used likelihood ratio tests to determine the significance of sex-by-genotype, sex-by-haplotype, and MDM2-by-MDM4 SNP interactions, overall and stratified by sex.

Patients who entered the study with a confirmed single primary melanoma and developed a second primary melanoma during the follow-up period (‘cross over’ cases, n = 96) were included as both cases and controls in the analysis for risk.

Cox proportional hazards models estimated hazard ratios (HRs) and 95% CIs to examine the effect of each allele, each inferred haplotype, and MDM2-MDM4 diplotype on melanoma-specific survival. Analysis of survival in SPM and MPM considered the time elapsed from the diagnosis of the index melanoma. Analyses were conducted in all participants, or overall cohort, and in subgroups stratified by sex. Baseline-adjusted models for each SNP accounted for age at diagnosis, sex, study center, and original SPM vs. MPM status. A time-dependent crossover variable was included to address the survival of individuals with single primaries who developed multiple primaries over the follow-up period. Fully adjusted models also accounted for the site of the tumor and log-transformed Breslow thickness, using the site and thickness of the thickest tumor for MPM cases. Sex-by-genotype, sex-by-haplotype, and MDM2-by-MDM4 SNP interaction analyses used the same baseline and fully adjusted models. The likelihood ratio test was used to test each interaction, with an *a priori* significance level of 0.20 [28,29,30]. The *a priori* SNPs were selected based on previous literature and findings; therefore, we did not apply multiple comparison adjustments due to the confirmatory nature of this study. All analyses were performed in the statistical package R v.3.1 and SAS 9.4 [31].

### 2.4. Assessment of Other Credible Risk Variants as Potential Candidates for the Study of Melanoma Risk and Outcomes

In exploratory analyses, we investigated *MDM2/MDM4* SNPs that are in strong LD with our tested variants, are likely to be functionally relevant based on their overlap with described DNA features and regulatory regions, and that have been characterized in relation to expression/transcription in the skin and in distal tissues from organs commonly targeted in melanoma metastases [32]. A similar approach was utilized in a large testicular cancer study [33]. Data for SNPs that met all three conditions were retrieved. Our rationale for this secondary analysis is based on two key points. First, the genetic environment (host or germline genetics) shaped by the *MDM2/MDM4* variants might have created a more favorable or unfavorable (depending on the alleles) tissue environment on the skin and/or in other organs, and thus the variants may modify the risk for developing subsequent (multiple) primary tumors, or progression through metastasis, and death of melanoma. Second, SNPs in high LD with our tested SNPs might be responsible for the *MDM2/MDM4* effects for risk and for survival observed in GEM. They might also explain discrepancies across studies–beyond the differences due to small sampling/low power to detect meaningful effects and/or lack of adjustment for covariates. For this approach, we identified SNPs in high LD with our tested *MDM2* and *MDM4* SNPs utilizing the LDlink suite [34,35]. Specifically, we used the LDproxy tool with a 100 Kb (+/− 50 Kb) window for each of the variants tested in GEM and retrieved SNPs with D’ ≥ 0.95 in White non-Hispanic populations (CEU, FIN, and GBR). SNPs were filtered according to their known and/or predicted overlaps with regulatory DNA elements using the RegulomeDB database [36,37], including SNPs ranked 1 (a through f) in RegulomeDB, as follows: *Rank 1a*, SNPs with supporting data as expression quantitative trait loci for genes (eQTL) AND have transcription factor (TF) binding AND match the TF binding motif AND match DNAse footprint and DNAse peak. *Rank 1b*, SNPs are eQTL AND have TF binding AND match any motif AND match DNAse footprint and DNAse peak. *Rank 1c*, eQTL AND have TF binding AND match TF motif + DNAse peak. *Rank 1d*, SNPs are eQTL AND have TF binding AND match any motif + DNAse peak. *Rank 1e*, SNPs are eQTL AND have TF binding AND match TF motif. *Rank 1f*, SNPs are eQTL AND have either TF binding or DNAse peak.

Data were retrieved using the LDexpress tool from the Genotype-Tissue Expression (GTEx) project’s portal to identify potential *MDM2/MDM4* SNPs with known effects on gene expression [38], focusing on statistically significant effects on transcription in the skin (presumed exposed or not exposed to sun based on anatomical location). Because organs and tissues prone to melanoma metastases include the lungs, liver, brain, and intestines, we also searched for potential associations with gene expression changes in these distal tissues.

## 3. Results

### 3.1. Study Population Characteristics

The characteristics of all study participants are described in Table 1. There is a higher proportion of males (56.5%) than females (43.5%), and the median age is 59.0 years (interquartile range (IQR) = 24.0 years). Most patients present with a thin melanoma ≤1.00 mm (61.6%) and one that is located on the trunk (43.3%). The females tend to be younger (median age = 53.0 years, IQR = 26.0 years) than males (median age = 64.0 years, IQR = 21.0 years) and have a greater proportion of tumors occurring on the extremities (56.9% vs. 24.1%) than does males. The median follow-up time is 7.6 years.

### 3.2. Effect of MDM2 and MDM4 Variants on Risk of Multiple Melanoma

Results from the analyses of the contribution of *MDM2* and *MDM4* gene variants to risk in 3616 GEM participants and in GEM participants stratified by sex, are presented in Table 2. No statistically significant associations were observed in the overall analysis after adjusting for age at diagnosis, sex, age-by-sex interaction, and study center between these gene variants and the likelihood of developing multiple melanomas. We observed a statistically significant genotype-by-sex interaction for *MDM4* rs4245739 (*p* = 0.01) and a borderline significant interaction for rs1563828 (*p* = 0.06). When we stratified the analysis by sex, a statistically significant increase in the likelihood of developing multiple melanomas was observed in females for each of the carried rs4245739-C alleles (OR_per-allele_ = 1.25, 95% CI 1.03–1.51, p-trend = 0.03). A similar but non-statistically significant increased likelihood for developing multiple melanomas was also observed in females carrying the *MDM4* T-C haplotype formed by rs1563828-rs4245739 (OR = 1.24, 95% CI = 1.02–1.50, and p_global_ = 0.09) (Table 3). No statistically significant interactions were observed between *MDM2* and *MDM4* SNPs (Table S6).

### 3.3. Associations between MDM2 and MDM4 Variants and Survival

Results from the analyses performed to investigate the contribution of *MDM2* and *MDM4* variants to melanoma-specific survival are shown in Table 4. There are 3521 total cases (1552 females and 1969 males) with available genotypes and follow-up data. Baseline models are presented in the Supplementary Material (Table S2). There are no statistically significant associations observed in the overall analysis for either *MDM2* or *MDM4* gene variants in models that included the covariates: age at diagnosis of the first primary, sex, study center, presence of multiple primary melanomas, and time-dependent crossover status for patients who entered the study with single primary melanoma and developed a subsequent melanoma during follow-up, anatomic site, and logged Breslow thickness of the deepest primary melanoma. In adjusted, stratified analyses by sex, the SNP309-G allele conferred a reduction in the risk of melanoma-specific death in females, which reached statistical significance: HR_per-allele_ = 0.63, 95% CI 0.42–0.95, and p_trend_ = 0.03. We conducted analyses in a subset of samples with complete staging information by including the variable stage in the Cox model (Table S3) and observed highly similar results to the analysis adjusted for logged Breslow thickness. To account for or rule out potential over-adjustment, we conducted a sensitivity analysis by excluding tumor thickness and observed no substantial differences in the results. We did not observe global differences in the distribution of haplotypes formed between rs117039649/SNP285 and rs2279744/SNP309 (p_global_ = 0.12), but improved survival was observed for females carrying the *MDM2* G-G haplotype: HR= 0.62 and 95% CI 0.40–0.94 (Table 5). Table S4 shows results obtained from a ‘minimally adjusted’ model in which tumor anatomic site and Breslow thickness were not included.

No other statistically significant associations were observed between the tested *MDM2* and *MDM4* variants and multiple melanoma or death in melanoma patients. Borderline significance was identified for the interaction between *MDM4* rs4245739 * *MDM2* rs117039649 on melanoma-specific survival (P_interaction_ 0.14, Table S6). Among men, there was a trend towards better survival for those carrying the rs117039649(GG)-*MDM4* rs4245739(AC) diplotype: HR= 0.72, 95% CI 0.52–1.00 (P_interaction_ 0.09, Table S7). No other significant interactions were observed.

### 3.4. Other MDM2 and MDM4 Candidate Loci for the Study of Melanoma Risk and Outcomes

We retrieved 1122 unique SNPs in high LD (D’ ≥ 0.95) with our tested SNPs. After excluding non-validated SNPs, 35 of the remaining 1117 SNPs are ranked 1a to 1f in RegulomeDB [30]: these SNPs are eQTLs and, at minimum, overlap TF binding sites and/or matched DNAse peaks. Of these, 24 have the highest likelihood of being co-inherited with our test SNPs (D’ ≥ 0.95). Setting a significance level for single tissue at *p*-values < 1 × 10^−4^ and multi-tissue posterior probability, m-values ≥ 0.9–1.0, we retrieved 16 functional SNPs that are in high LD with the tested variants in the GEM cohort (D’ > 0.98, and for 10 SNPs, R^2^ ≥ 0.8) and that modify gene transcription in skin and/or distal tissues. Our findings are based on data available for up to 605 sun-exposed and 517 non-exposed skin tissues, 515 lung tissues, and a range of 126 to 405 brain tissues, depending on the tested gene, as detailed in Table S5A,B. No significant associations are found with expression in intestinal epithelia or liver tissue, and no data are available for gene expression in bone.

For the *MDM2* SNPs, the effect sizes on gene expression of the solute carrier family 35 member E3 gene (*SLC35E3*) in brain tissue ranges from −0.33 to −0.504, *p*= 3 × 10^−6^ to 4 × 10^−17^). Three SNPs affect the expression of the nucleoporin 107 gene (*NUP107*) in the skin (effect size from 0.119 to 0.176, *p* = 2 × 10^−5^ to 5 × 10^−11^) and in the lung (effect size 0.132, *p* = 1 × 10^−6^) tissues. For SNPs in high LD with the tested *MDM4* SNPs, we find eight associations with lower expression of *MDM4*, mainly in the lung (effect size from −0.12 to −0.135, *p* = 2 × 10^−4^ to 3 × 10^−6^) and seven SNPs with downregulation in brain tissues (effect size from −0.246 to −0.271, *p* = 2 × 10^−5^ to 9 × 10^−6^). Expression of the Phosphatidylinositol-4-Phosphate 3-Kinase Catalytic Subunit Type 2 Beta gene (*PIK3C2B*) appears downregulated in skin tissues (effect size from −0.103 to −0.176, *p*= 2 × 10^−5^ to 9 × 10^−11^). Two SNPs are positively associated with the expression of the long noncoding RNA (lncRNA) RP11-430C7.5 in the lung (effect size from 0.152–0.158, *p* = 2 × 10^−5^ to 4 × 10^−5^) (Table S5B).

## 4. Discussion

This study evaluated potential associations between a priori functionally relevant SNPs in *MDM2* and *MDM4* and multiple primary vs. single primary melanoma development, as well as risk for melanoma-specific death, within a large population-based cohort of melanoma cases for which well-annotated demographics, clinicopathologic, melanoma covariates, and follow up data were available. We identified differential gene associations in relation to risk and survival by sex. Specifically, females, but not men, with the *MDM4* rs4245739*C allele were more likely to develop multiple primaries, and those with the *MDM2* SNP309*G allele were less likely to succumb to melanoma compared with those carrying the C allele. Considering the increased affinity of Sp1 for its binding site in position 309 of the *MDM2* promoter when G is present and that the G-allele has been linked to increased *MDM2* mRNA and Mdm2 protein [14,15], and that higher levels of MDM2 predict better outcomes in melanoma [39], our findings of a protective effect in carriers of *MDM2* SNP309*G are biologically plausible. We can also postulate that in women, upon hormonal stimulation, estrogen receptor-alpha (ER-α) and Sp1 are recruited to the *MDM2* promoter [40,41], potentiating further the effect of the SNP309-G allele. We anticipated observing a greater effect with the *MDM2* haplotype, but the associations were not significant (global *p*-value = 0.12). Two of the haplotypes (SNP285-SNP309 C-T and C-G) were too infrequent (≤3%); however, the protective effect of the SNP309-G can be noticed within the G-G haplotype. This was unopposed by the effect of the C-allele, thought to exert a greater effect in the opposite direction, potentially in a tissue-dependent manner [17], as most associations have been reported in gynecological cancers [42].

In our previous investigation of SNP309 in a hospital-based cohort of nearly 1000 melanoma patients, the *MDM2* SNP309 allele did not have a statistically significant effect on disease recurrence or melanoma death; however, in adjusted analysis for covariates, we did observe a trend suggestive of a protective effect for the SNP309*G-allele in relation to mortality: HR_het_ = 0.74, 95% CI 0.47–1.15, and HR_GG_= 0.59, 95% CI 0.32–1.10 [13]. It is plausible that Mdm2 alone or in concert with Mdm4 plays an important role through p53 -dependent and independent circuits [18,19,43,44,45]. In fact, we observed a borderline significant interaction between *MDM2* rs117039649 (SNP285)–*MDM4* rs4245739, but a greater number of cases is necessary to confirm these observations. If the p53-Mdm2 interaction indeed exerts an important role in melanoma, targeted therapy might be considered in the future. For example, a strategy could involve blocking the interaction between wild-type p53 and Mdm2, or de-stabilizing the MDM2-MDM4 heterocomplex, especially given that the currently available small molecule Mdm2 inhibitors have low affinity for Mdm4 [46,47].

The *MDM4* SNP rs4245739 overlaps with a putative miRNA target site on the 3′ UTR, greatly increasing the affinity for miR-191-5p when rs4245739*C is present [20,48]. Interestingly, miR-191 is responsive to estrogen [49,50]; thus, one would anticipate that females expressing more miR-191 would have further inhibited *MDM4* expression. This idea is supported by reports of a reduced risk of esophageal cancer in females and an increased risk of estrogen-negative breast cancer in C-allele carriers [51,52]. A meta-analysis of 15 studies and over 69,000 subjects found an overall inverse association between *MDM4* rs4245739*C and risk for breast, ovarian, endometrial, lung, colon, esophageal cancer, prostate, and non-Hodgkin’s lymphoma [53]. Notably, in this meta-analysis, the positive association with risk seemed driven by the collective effect of the smaller studies in Asians [53], including a more recent study [54], while the larger studies in Caucasians had the opposite [52] or null [55,56] effects. The in-depth relationship of miR-191 binding and *MDM4* in relation to melanoma development remains to be elucidated, adding in some way to the inconclusive research on the role of hormonal and reproductive factors in melanoma to date [57]. *MDM4*-rs4245739 is in strong LD with rs1563828 (D’ = 1, r2 = 0.8397) and with a number of variants located in an evolutionarily conserved haplotype. Here, we found no associations between *MDM4*-rs1563828 and risk in the GEM cohort. In a study that included 258 sporadic and 50 familial melanoma cases and 799 unaffected controls, Thunell and colleagues (2014) investigated this variant and reported a decreased risk of sporadic melanoma in heterozygote males (rs1563828-C/T), OR = 0.54, 95% CI 0.34–0.85, *p*-value = 0.01) compared with the CC genotype [16]. Notably, though, the effect was only observed in a small sample and was not replicated in familial cases [16].

To identify credible SNPs responsible for, or contributing to, the effect of the tested *MDM2/MDM4* SNPs on melanoma, we mined public databases. We found several variants in perfect or very high LD with our tested SNPs that appear to modulate the expression of three genes and a long noncoding RNA in the skin and distal tissues. For instance, the *MDM2* rs2120742*T allele upregulates expression of the *NUP107* gene in the skin from the lower leg, which is presumably sun exposed. The nucleoporin 107 protein participates in interferon signaling and mitotic prophase, and a homozygous mutation in the *NUP107* gene causes ovarian dysgenesis in females [58] with no effect on the development of male gonads. Interestingly, a variant in *NUP107* has been reported to be significantly associated with resistance to chemotherapy in ovarian cancer [59]. Overexpression of *NUP107* in cervical tumors has been reported, and it confers a pro-survival advantage through resistance to oxidative damage that can be reversed by silencing *NUP107* [60]. In non-small cell lung cancer, *NUP107* overexpression has also been previously reported [61], and another nucleoporin family member, NUP37, was found responsible for increased cell proliferation in vitro [62]. The *MDM4* SNP rs4951382 is coinherited with our tested SNPs, and the C-allele is linked to overexpression of RP11-430C7.5 in the lung (Table S5A,B), but little is known about RP11-430C7.5, except for its reported association with pancreatic ductal adenocarcinoma prognosis [63].

To our knowledge, ours is the first study to date to investigate the effect of the two known functional *MDM4* and *MDM2* variants on the risk of developing subsequent primaries (as a proxy for risk for melanoma) and melanoma-specific survival and the first to identify sex-dependent associations in a large population-based cohort of melanoma cases. One limitation of this study was the low frequency of some alleles, which may have affected the power to uncover meaningful associations among the haplotypes. Functional assays in melanoma cells to better understand the observed associations with the tested SNPs were beyond the scope of the current work. We set very stringent criteria for retrieving functional SNPs based on the highest RegulomeDB rank (1a–1f), and eQTL were retrieved based on the most significant associations with skin and tissues prone to ‘hosting’ metastatic melanoma; therefore, it is likely that additional *MDM2/MDM4* SNPs not yet identified are relevant for melanoma development and/or progression. This study involved patients of European descent, and our results may not apply to other populations; however, cutaneous melanoma is much more prevalent in non-Hispanic Whites. Similarly, the death rate attributed to melanoma is much higher in non-Hispanic White people compared with Hispanic, Black, American Indian/Alaska natives, or Asian/Pacific Islanders. Key strengths include the large number of cases which provided a well-powered study. In addition, the international and population-based nature of our sample allows our data to be generalizable to other populations of European descent, and, due to our unique case-control design, results from our analyses investigating the risk of multiple melanomas compared with single melanoma can be extrapolated to overall melanoma risk. Further, the inclusion of both single and multiple primary cases enabled investigations into both risk and survival in the same cohort. Importantly, rigorously collected data were available on covariates relevant for both risk and survival analyses, limiting the possibility of biased results.

## 5. Conclusions

Our data provide some evidence for sex-specific associations of *MDM4*-rs4245739 and *MDM2*-rs2279744 (SNP309) with melanoma risk and survival, respectively. Several functional *MDM2/MDM4* SNPs in high LD with variants tested in our melanoma cohort modulate expression in skin and in distal organs—to where melanoma often spreads—affecting molecules that have been previously associated with tumor progression, resistance to chemotherapy, and prognosis in gynecological cancers, among others. Thus, it is plausible that these credible variants in *MDM2* and *MDM4* modify the risk for melanoma and survival in melanoma patients. Our findings in this large population-based melanoma study now pave the way for further replication studies and for research to unveil the potential underlying mechanisms involving Mdm2/Mdm4 with melanoma development and outcomes.

## Figures and Tables

**Table 1:** Characteristics of participating melanoma cases and tumors.

Characteristic ^a^	Total N (%)	N Females (%)	N Males (%)
**Melanoma participants**	3663	1595 (43.5)	2068 (56.5)
**Age at diagnosis in years, median (IQR)**			
	53.0 (26.0)	53.0 (26.0)	64.0 (21.0)
<40	348 (21.8)	348 (21.8)	161 (7.8)
40–49	328 (20.6)	328 (20.6)	244 (11.8)
50–59	326 (20.4)	326 (20.4)	430 (20.8)
60–69	245 (15.4)	245 (15.4)	504 (24.4)
70–79	263 (16.5)	263 (16.5)	555 (26.8)
>80	85 (5.3)	85 (5.3)	174 (8.4)
**Multiple vs. single primary melanoma status**			
Single primary	1184 (74.2)	1184 (74.2)	1274 (61.6)
Multiple primaries	411 (25.8)	411 (25.8)	794 (38.4)
**Breslow thickness in mm, median (IQR)**			
	0.60 (0.65)	0.60 (0.65)	0.65 (0.90)
In situ	114 (7.2)	114 (7.2)	187 (9.0)
0.01–1.00	1056 (66.2)	1056 (66.2)	1202 (58.1)
1.01–2.00	235 (14.7)	235 (14.7)	366 (17.7)
2.01–4.00	114 (7.2)	114 (7.2)	165 (8.0)
>4.00	43 (2.7)	43 (2.7)	103 (5.0)
Missing	33 (2.1)	33 (2.1)	45 (2.2)
**Anatomic site**			
Head/neck	212 (13.3)	212 (13.3)	460 (22.2)
Trunk	476 (29.8)	476 (29.8)	1110 (53.7)
Upper extremities	365 (22.9)	365 (22.9)	303 (14.7)
Lower extremities	542 (34.0)	542 (34.0)	195 (9.4)

Abbreviations: N, number; IQR, interquartile range; and mm: millimeters. ^a^ Age corresponds to the diagnosis of the most recent primary melanoma; Breslow tumor thickness corresponds to the thickest tumor in cases with multiple primary melanomas; and anatomic site refers to the index melanoma (thickness of the second or subsequent primary in cases with multiple melanomas).

**Table 2:** Associations between *MDM2* and *MDM4* SNPs and multiple melanomas by sex in logistic regression models adjusted for melanoma covariates.

	All Cases (N = 3616)		Females (N = 1583)		Males (N = 2033)	
Gene Variants, Major/Minor Alleles	OR (95% CI) ^a^	P_trend_^a^	OR (95% CI) ^c^	P_trend_^c^	OR (95% CI) ^c^	P_trend_^c^
***MDM2***						
rs117039649 (SNP285), G/C	0.99 (0.75–1.30)	0.92	1.21 (0.79–1.85)	0.37	0.87 (0.60–1.25)	0.44
rs2279744 (SNP309), T/G	1.04 (0.93–1.16)	0.52	1.00 (0.84–1.18)	0.99	1.06 (0.92–1.23)	0.40
P_interaction_ = 0.24 (rs117039649) and 0.61 (rs2279744) ^b^						
***MDM4***						
rs1563828, C/T	0.99 (0.88–1.11)	0.81	1.13 (0.94–1.36)	0.19	0.90 (0.77–1.04)	0.16
rs4245739, A/C	1.03 (0.91–1.16)	0.69	1.25 (1.03–1.51)	**0.03**	0.90 (0.77–1.05)	0.18
P_interaction_ = **0.06** (rs1563828) and **0.01** (rs4245739) ^b^						

Abbreviations: N, number; OR, Odds Ratio; and CI, Confidence Interval. P_trend_, trend *p*-values, and P_interaction_, the *p*-value for the interaction term. ^a^ Logistic regression model adjusted for age at diagnosis, sex, age*sex interaction, and study center; per-allele OR, CI, and trend *p*-values are for carriage of minor alleles (additive model). ^b^ Likelihood ratio test *p*-value for sex*genotype interaction term. ^c^ Logistic regression model adjusted for age at diagnosis and study center; per-allele OR, CI, and trend *p*-values are for carriage of minor alleles (additive model). Significant associations are shown in bold type. Number of available genotypes are MDM2-rs117039649 (SNP285), n = 3616 and rs2279744 (SNP309), n = 3457; and MDM4-rs1563828, n = 3575 and rs4245739, n = 3574. Number of multiple primary melanoma cases are *MDM2*-rs117039649 (SNP285), n = 1195 and rs2279744 (SNP309), n = 1139; and *MDM4*-rs1563828, n = 1180 and rs4245739, n = 1185.

**Table 3:** Association between *MDM2* and *MDM4* haplotypes and multiple melanomas by sex in logistic regression models adjusted for melanoma covariates.

	All (N = 3663)			Females (N = 1595)		Males (N = 2068)	
Haplotype	Frequency	OR (95% CI) ^a^	P_global_^a^	OR (95% CI) ^c^	P_global_^c^	OR (95% CI) ^c^	P_global_^c^
MDM2							
rs117039649-rs2279744 (SNP285-SNP309)							
G–T	0.64	1 (ref)		1 (ref)		1 (ref)	
G–G	0.32	1.02 (0.91–1.14)		0.985 (0.83–1.18)		1.034 (0.89–1.20)	
C–T	0.01	0.50 (0.21–1.20)		1.717 (0.52–5.72)		0.152 (0.03–0.68)	
C–G	0.03	1.12 (0.82–1.54)	0.42	1.12 (0.69–1.82)	0.75	1.156 (0.77–1.75)	0.09
P_interaction_ = 0.08 ^b^							
MDM4							
rs1563828-rs4245739							
C–A	0.68	1 (ref)		1 (ref)		1 (ref)	
T–C	0.26	1.03 (0.91–1.17)	0.20	1.24 (1.02–1.50)	0.09	0.91 (0.78–1.08)	0.31
C–C	0.01	0.76 (0.44–0.31)		0.72(0.20–2.56)		0.73 (0.40–1.34)	
T–A	0.05	0.79 (0.61–1.02)		0.80 (0.53–1.19)		0.78 (0.55–1.09)	
P_interaction_ = 0.15 ^b^							

Abbreviations: N, number; OR, Odds Ratio; CI, Confidence Interval; P_global_, global *p*-value; P_interaction_, *p*-value for interaction term; and ref, referent group. ^a^ Logistic regression model adjusted for age at diagnosis, sex, age*sex interaction, and study center. ^b^ Likelihood ratio test *p*-value for sex*haplotype interaction term in the model with all participants. ^c^ Model also adjusted for age at diagnosis and study center. Global *p*-value corresponds to the simultaneous comparison of each haplotype combination to the reference haplotype.

**Table 4:** Effect of *MDM2* and *MDM4* gene variants on risk for melanoma-specific death by sex in models fully adjusted for melanoma covariates.

All (N = 3521)				Females (N = 1552)			Males (N = 1969)		
Gene Variants, Major/Minor Alleles	N Total/N Deaths ^a^	HR (95% CI) ^b^	P_trend_^b^	N Total/N Deaths ^a^	HR (95% CI) ^d^	P_trend_^d^	N Total/N Deaths ^a^	HR (95% CI) ^d^	P_trend_^d^
***MDM2***									
rs117039649 (SNP285), G/C	3521/248	0.65 (0.38–1.12)	0.12	1552/70	0.68 (0.27–1.69)	0.41	1969/178	0.61 (0.31–1.21)	0.16
rs2279744 (SNP309), T/G	3367/236	0.84 (0.69–1.02)	0.07	1484/67	0.63 (0.42–0.95)	**0.03**	1883/169	0.94 (0.75–1.17)	0.57
P_interaction_ = 0.77 (rs117039649) and **0.13** (rs2279744) ^c^									
***MDM4***									
rs1563828, C/T	3484/245	0.92 (0.75–1.12)	0.39	1541/70	1.09 (0.77–1.55)	0.63	1943/175	0.84 (0.66–1.07)	0.16
rs4245739, A/C	3480/250	0.89 (0.72–1.1)	0.28	1532/70	1.03 (0.71–1.50)	0.89	1948/180	0.82 (0.64–1.06)	0.12
P_interaction_ = 0.25 (rs1563828) and 0.33 (rs4245739) ^c^									

Abbreviations: N, number; HR, Hazard Ratio; CI, Confidence Interval; P_trend_, trend *p*-values; P_interaction_, and *p*-value for the interaction term. ^a^ N total, all participants; N deaths, number of melanoma-specific deaths. ^b^ Per allele HR, 95% CI, and *p*-values obtained from a Cox proportional hazards model adjusted for age at diagnosis of the first primary melanoma, sex, study center, single or multiple primary melanoma status, time-dependent crossover status (for patients who entered the study with single primary melanoma and developed a subsequent melanoma during follow up), anatomic site and logged Breslow thickness of the deepest primary melanoma. ^c^ Likelihood ratio test *p*-value for sex*SNP interaction term. ^d^ Per allele HR, 95% CI, and *p*-values obtained from fully adjusted models in stratified analysis by the female or male sex. Significant associations are shown in bold type (*p* < 0.05 for trend *p*-values and <0.20 for interaction *p*-values).

**Table 5:** Effect of *MDM2* and *MDM4* haplotypes on risk for melanoma-specific death by sex in models adjusted for melanoma covariates.

All (N = 3521)				Females (N = 1552)			Males (N = 1969)		
Haplotype	Frequency	HR (95% CI) ^a^	P_global_ ^a^	Frequency	HR (95% CI) ^c^	P_global_ ^c^	Frequency	HR (95% CI) ^c^	P_global_ ^c^
***MDM2***									
rs117039649-rs2279744 (SNP285-SNP309)									
G-T	0.642	1 (ref)		0.639	1 (ref)		0.644	1 (ref)	
G-G	0.322	0.86 (0.71–1.06)		0.325	0.62 (0.40–0.94)		0.320	0.98 (0.78–1.24)	
C-T	0.006	0.88 (0.23–3.32)		0.006	0.17 (0–11.25)		0.006	1.45 (0.35–6.10)	
C-G	0.030	0.55 (0.28–1.08)	0.18	0.030	0.72 (0.25–2.05)	0.12	0.030	0.45 (0.18–1.10)	0.38
P_interaction_ = 0.21 ^b^									
***MDM4***									
rs1563828-rs4245739									
C-A	0.679	1 (ref)		0.682	1 (ref)		0.677	1 (ref)	
T-C	0.262	0.93 (0.75–1.15)	0.56	0.261	1.07 (0.73–1.56)		0.263	0.87 (0.67–1.12)	
T-A	0.050	0.88 (0.57–1.37)		0.052	1.28 (0.62–2.62)		0.049	0.75 (0.42–1.32)	
C-C	0.009	0.02 (0–15.83)		0.006	0.02 (0–31.02)	0.87	0.011	0.03 (0–26.12)	0.34
P_interaction_ = 0.67 ^b^									

Abbreviations: N, number; HR, Hazard Ratio; CI, Confidence Interval; P_global_, global *p*-value; P_interaction_, the *p*-value for interaction term; and ref, reference group. ^a^ Per-haplotype subdistribution; HR, CI, and global *p*-values obtained from a Cox proportional hazards model adjusted for age at diagnosis, sex, study center; single or multiple primary melanoma status plus a time-dependent variable (for cases with single primary melanoma who developed a subsequent melanoma during follow-up), anatomic site and logged Breslow thickness of the deepest primary melanoma. The reference group corresponds to the most common haplotype. ^b^ Likelihood ratio test *p*-value for sex*haplotype interaction term. ^c^ Per-haplotype HR, CI, and global *p*-values from fully adjusted models (as per footnote ‘a’, except sex), in analysis stratified by sex.

## Data Availability

Data presented in this study are available upon request from the corresponding author and upon approval by the GEM Study Steering Committee, as well as clearance with the IRB.

## Supplementary Materials

The following supporting information can be downloaded at: https://www.mdpi.com/article/10.3390/cancers15102707/s1, Table S1: Characteristics of *MDM2* and *MDM4* gene variants studied in GEM; Table S2: Minimally adjusted hazard ratios for *MDM2* and *MDM4* gene variants on the risk for melanoma-specific death in GEM; Table S3: Hazard ratios for MDM2 and MDM4 gene variants on the risk for melanoma-specific death in GEM by sex, adjusted for stage (subanalyses); Table S4: Baseline adjusted models for association between *MDM2* and *MDM4* haplotypes and melanoma-specific death by sex; Table S5A: Characteristics of other credible variants, SNPs co-inherited with the MDM2/MDM4 SNPs tested in GEM that modulate gene transcription in unaffected skin, and distal organs/tissues; Table S5B: Effect of other credible MDM2 and MDM4 variants on transcription in unaffected skin, and in distal organs/tissues often targeted during melanoma metastases; Table S6: Interaction between MDM2 and MDM4 SNPs on risk for developing multiple melanoma, and risk of melanoma-specific death; Table S7: Minimally adjusted hazard ratios for MDM2 and MDM4 diplotypes and melanoma-specific death by sex.
