# Supplementary material for: Sex-Specific Associations of MDM2 and MDM4 Variants with Risk of Multiple Primary Melanomas and Melanoma Survival in Non-Hispanic Whites
Source: Cancers (Basel). 2023 May 11;15(10):2707. doi: 10.3390/cancers15102707 (PMC10216616; doi:10.3390/cancers15102707)
Supplement: Supplementary file 1 [file cancers-15-02707-s001.zip › cancers-2354710-Supplementary Materials.pdf]

Supplementary Materials: Sex-Specific Associations of *MDM2* and *MDM4* Variants with Risk of Multiple Primary Melanomas and Mela-Noma Survival in Non-hispanic Whites

Sarah V. Ward, Isidora Autuori, Li Luo, Emily LaPilla, Sarah Yoo, Ajay Sharma, Klaus Busam, David Olilla, Terence Dwyer, Hoda Anton-Culver, Roberto Zanetti, Lidia Stacchetto, Anne E. Cust, Richard P. Gallagher, Peter A. Kanetsky, Stefano Ross, Colin B.F Begg, Marianne Berwick, Nancy E. Thomas and Irene Orlow, on behalf of the GEM Study Group

Table S1. Characteristics of MDM2 and MDM4 gene variants investigated in the Genes, Environment and Melanoma study.

Gene variants	D' [a]	R^2 [a]	N[b]	Position (GRCh38.p13)	A/a[c]	Relative position	MAF in GEM	MAF reported[d]	RegDB rank[e]	RegDB score[e]
MDM2										
rs117039649 (SNP285)	1	0	3616	Chr12:68808776	G/C	intronic	0.04	0.0349	2a	0.419
rs2279744 (SNP309)			3457	Chr12:68808800	T/G	intronic	0.35	0.3065	4	0.705
MDM4										
rs1563828	1	0.7	3575	Chr1:204547449	G/A	intronic	0.31	0.3286	6	0.194

Abbreviations: GEM, Genes, Environment and Melanoma Study; N, number of samples with genotype data; A/a, Major/minor alleles; MAF, minor allele frequency; RegDB, Regulome database. [a] D' and r2 retrieved from NCI's LD Link (https://ldlink.nci.nih.gov/) for White non-Hispanic population (CEU, TSI, FIN, GBR). [b] N samples passing QC. [c] Major and minor alleles noted for the forward or plus strand; the designed assay typed the reverse or minus strand of rs1563828 (i.e., C>T). [d] dbSNP, ALFA project or gnomAD in European population. [e] RegulomeDB (https://regulomedb.org/regulome-search/). All public databases accessed last in February 2023.

Table S2. Minimally adjusted hazard ratios for *MDM2* and *MDM4* gene variants on the risk for melanoma-specific death in GEM by sex.

	**All (n =3521)**				**Females (n =1552)**				**Males** (n =1969)			
**Gene and RefSeq**	**N total / N deaths[a]**	**HR[b]**	**95% CI[b]**	P_{trend}[b]	**N total / N deaths[a]**	**HR[d]**	**95% CI[d]**	P_{trend}[d]	**N total / N deaths[a]**	**HR[d]**	**95% CI[d]**	P_{trend}[d]
MDM2												
rs117039649 (SNP285)	3521/248	0.70	0.41 - 1.21	0.21	1552/70	0.91	0.38 - 2.18	0.83	1969/178	0.62	0.31 - 1.24	0.18
rs2279744 (SNP309)	3367/236	0.89	0.74 - 1.08	0.24	1484/67	0.73	0.51 - 1.06	0.10	1883/169	0.97	0.78 - 1.21	0.80
$P_{interaction}$=0.47 (rs117039649) and 0.26 (rs2279744) [c]												
MDM4												
rs1563828	3484/245	0.90	0.74 - 1.10	0.30	1541/70	1.12	0.78 - 1.61	0.56	1943/175	0.82	0.65 - 1.05	0.11
rs4245739/SNP34091	3480/250	0.88	0.72 - 1.08	0.22	1532/70	1.07	0.73 - 1.57	0.72	1948/180	0.81	0.64 - 1.04	0.10
$P_{interaction}$=0.20 (rs1563828) and 0.26 (rs4245739) [c]												

Abbreviations: N, number; HR, Hazard Ratio; CI, Confidence Interval; P_{trend}, trend p-values; $P_{interaction}$, p-value for interaction term.[a] N total, all participants; N deaths, number of melanoma-specific deaths. [b] Per allele HR, 95% CI, and p-values obtained from a Cox proportional hazards model adjusted for age at diagnosis of the first primary melanoma, sex, study center, single or multiple primary melanoma status, time dependent crossover status (for patients who entered the study with single primary melanoma and developed a subsequent melanoma during follow up). [c] Likelihood ratio test p-value for sex*SNP interaction term. [d] Per allele HR, 95% CI, and p-values obtained in stratified analysis by female or male sex.

Table S3. Hazard ratios for *MDM2* and *MDM4* gene variants on the risk for melanoma-specific death in GEM by sex, adjusted for Stage (subanalyses).

SNP and cohort		N total / N deaths[a]	HR[b]	95% CI[b]	P_{value}[b]	HR[c]	95% CI[c]	P_{value}[c]
	All	2614/ 193	0.71	0.40, 1.23	0.22	0.77	0.44, 1.37	0.38
rs117039649	Females	1160/ 56	0.62	0.22, 1.72	0.35	0.89	0.32, 2.47	0.83
	Males	1454/ 137	0.72	0.37, 1.41	0.34	0.72	0.36, 1.45	0.36
	All	2494/ 185	0.8	0.64, 1.00	0.05	0.8	0.64, 0.99	0.04
rs2279744	Females	1108/ 53	**0.57**	**0.36, 0.92**	**0.02**	**0.55**	**0.34, 0.88**	**0.01**
	Males	1386/ 132	0.89	0.70, 1.15	0.38	0.89	0.69, 1.15	0.36
	All	2596/ 193	0.92	0.74, 1.15	0.47	0.9	0.72, 1.12	0.35
rs1563828	Females	1154/ 56	1.08	0.72, 1.62	0.71	1.12	0.73, 1.71	0.61
	Males	1442/ 137	0.86	0.67, 1.12	0.27	0.83	0.64, 1.08	0.16
	All	2581/ 196	0.88	0.70, 1.11	0.30	0.86	0.68, 1.08	0.20
rs4245739	Females	1141/ 56	0.99	0.64, 1.54	0.97	1.03	0.65, 1.62	0.91
	Males	1440/ 140	0.84	0.64, 1.10	0.20	0.8	0.61, 1.05	0.11

Abbreviations: N, number; HR, Hazard Ratio; CI, Confidence Interval. [a] N total, all participants with available Stage; N deaths, number of melanoma-specific deaths. [b] Per allele HR, 95% CI, and p-values obtained from a Cox proportional hazards model adjusted for age at diagnosis of the first primary melanoma, sex, study center, single or multiple primary melanoma status, time-dependent crossover status (for patients who entered the study with single primary melanoma and developed a subsequent melanoma during follow up), anatomic site and logged Breslow thickness of the deepest primary melanoma. [c] Per allele HR, 95% CI, and p-values obtained from a Cox proportional hazards model adjusted for age at diagnosis of the first primary melanoma, sex, study center, single or multiple primary melanoma status, time-dependent crossover status, anatomic site and stage, considering the deepest primary melanoma.

Table S4. Minimally adjusted hazard ratios for *MDM2* and *MDM4* haplotypes and melanoma-specific death by sex.

	**All (n=3663)**				**Females (n=1595)**				**Males** (n=2068)			
**Haplotype**	**Frequency**	**HR**[a]	**95% CI**[a]	P_{global}[a]	**Frequency**	**HR**[c]	**95% CI**[c]	P_{global}[c]	**Frequency**	**HR**[c]	**95% CI**[c]	P_{global}[c]
MDM2												
**rs117039649 - rs2279744** (SNP285-SNP309)												
G-T	0.64	1.00	(ref)		0.64	1.00	(ref)		0.64	1.00	(ref)	
G-G	0.32	0.92	0.76 - 1.12		0.33	0.71	0.49 - 1.05		0.32	1.02	0.82 - 1.28	
C-G	0.03	0.60	0.31 - 1.14		0.03	1.00	0.37 - 2.71		0.03	0.47	0.20 - 1.11	
C-T	0.01	1.09	0.30 - 3.94	0.39	0.01	0.28	0.01 - 14.74	0.36	0.01	1.69	0.43 - 6.65	0.33
$P_{interaction}$=0.24[b]												
MDM4												
**rs1563828 - rs4245739**												
All												
C-A	0.68	1.00	(ref)		0.68	1.00	(ref)		0.68	1.00	(ref)	
T-C	0.26	0.92	0.74 - 1.13		0.26	1.12	0.76 - 1.65		0.26	0.84	0.66 - 1.08	
T-A	0.05	0.87	0.56 - 1.35		0.05	1.15	0.56 - 2.38		0.05	0.77	0.44 - 1.33	
C-C	0.01	0.03	0 - 18.27	0.52	0.01	0.01	0 - 3.25×10^9	0.92	0.01	0.026	0 - 29.0	0.30
$P_{interaction}$=0.64[b]												

Abbreviations: N, number; HR, Hazard Ratio; CI, Confidence Interval; P_{global}, global p-value; $P_{interaction}$, p-value for interaction term; ref, reference group. [a] Per-haplotype subdistribution; HR, CI, and global p-values obtained from a Cox proportional hazards model adjusted for age at diagnosis, sex, study center, single or multiple primary melanoma status, plus a time-dependent variable for cases with single primary melanoma who developed a subsequent melanoma during follow-up. The referent group corresponds to the most common haplotype. [b] Likelihood ratio test p-value for sex*haplotype interaction term. [c] Per-haplotype HR, CI, and global p-values in models adjusted for age at diagnosis, center, single or multiple primary melanoma status, and time-dependent variable for cases with single primary melanoma who developed a subsequent melanoma during follow-up (as per footnote 'a', except sex), in analysis stratified by sex.

Table S5. A. Characteristics of other credible variants: SNPs co-inherited *with the MDM2/MDM4* SNPs tested in GEM that modulate gene transcription in unaffected skin, and distal organs/tissues.

RefSeq_Test[a]	SNP in LD with Tests[b]	D'[b]	R2[b]	Gene : Consequence	RegDB Rank[c]	RegDB Score[c]
MDM2						
rs2279744	rs1201644	1	0.591	MDM2 Splice donor	1f	0.55436
	rs1625525	1	0.487	MDM2: Intron	1f	0.55436
	rs2120742	1	0.454	LOC100130075: Non coding transcript exon	1f	0.55436
MDM4						
rs1563828	rs12041243	1	0.129	MDM4: Intron	1b	0.51695
rs1563828	rs12039365	1	0.129	MDM4: Intron	1f	0.55324
rs4245739	rs4951407	0.979	0.682	LOC105371692: Regulatory region	1f	0.55436
rs4245739	rs4951407	0.979	0.682	LOC105371692: Regulatory region	1f	0.55436
rs1563828 & rs4245739	rs1380576	1	0.95	MDM4: Intron	1b	0.99621
rs1563828 & rs4245739	rs1380576	1	0.95	MDM4: Intron	1b	0.99621
	rs4951393	1	0.992	MDM4: Intron	1d	0.64833
	rs4951393	1	0.992	MDM4: Intron	1d	0.64833
	rs10494852	0.991	0.925	PIK3C2B: Intron	1f	0.22271
	rs10494852	0.991	0.925	PIK3C2B: Intron	1f	0.22271
	rs10900594	0.991	0.917	Intergenic	1f	0.22271
	rs10900594	0.991	0.917	Intergenic	1f	0.22271
	rs12039454	1	1	TF binding site	1f	0.55436
	rs12039454	1	1	TF binding site	1f	0.55436
	rs16853958	1	0.926	Intergenic	1f	0.55324
	rs16853958	1	0.926	Intergenic	1f	0.55324
	rs2369244	1	0.992	MDM4: Intron	1f	0.55436
	rs2369244	1	0.992	MDM4: Intron	1f	0.55436
	rs4252725	1	0.926	MDM4: Intron	1f	0.60183
	rs4252725	1	0.926	MDM4: Intron	1f	0.60183
	rs4951382	0.99	0.792	PIK3C2B: Intron	1f	0.55436
	rs4951382	0.99	0.792	PIK3C2B: Intron	1f	0.55436
	rs7556371	0.99	0.815	PIK3C2B: Intron	1f	0.22271
	rs7556371	0.99	0.815	PIK3C2B: Intron	1f	0.22271

Abbreviations: RefSeq, reference sequence; SNP, single nucleotide polymorphism; LD, linkage disequilibrium; RegDB, RegulomeDB. [a] SNPs tested in the GEM study and in this study: rs2279744 in MDM2, rs1563828 and rs4245739 in MDM4. For the tested MDM2-rs117039649 no SNPs in LD matched our selection criteria in relation to functional relevance or effect on transcription in skin and target distal tissues. [b] SNPs in high LD with SNPs tested in GEM participants (D'>0.95). [c] RegulomeDB ranks 1a to 1f.

Table S5. B. Effect of other credible *MDM2*/*MDM4* variants on transcription in unaffected skin, and in distal organs/tissues often targeted during melanoma metastases.

SNP in LD with Tests [a]	Gene Symbol[b]	Effect Allele Freq.[b]	Skin not sun exposed[b]		sun exposed[b]		Brain spinal cord [b]		hippocampus [b]		substantia nigra [b]		cerebellum [b]		Lung [b]	
			Effect Size	P-value	Effect Size	P-value	Effect Size	P-value	Effect Size	P-value	Effect Size	P-value	Effect Size	P-value	Effect Size	P-value
MDM2																
rs1201644	SLC35E3	A=0.507					-0.443	$5x10^{-12}$	-0.33	$3x10^{-6}$	-0.348	$8x10^{-6}$				
	NUP107	A=0.507			0.119	$2x10^{-5}$										
rs1625525	SLC35E3	C=0.458					-0.49	$3x10^{-16}$	-0.33	$4x10^{-6}$	-0.397	$2x10^{-7}$				
	MDM2	C=0.458					-0.366	$2x10^{-7}$								
	NUP107	C=0.458			0.138	$3x10^{-7}$										
rs2120742	SLC35E3	T=0.441					-0.504	$4x10^{-17}$	-0.352	$5x10^{-7}$	-0.379	$3x10^{-7}$				
	MDM2	T=0.441					-0.383	$6x10^{-8}$								
	NUP107	T=0.441			0.176	$5x10^{-11}$									0.132	$1x10^{-6}$
MDM4																
rs12041243	PIK3C2B	G=0.242	-0.147	$4x10^{-6}$	-0.176	$9x10^{-11}$										
rs12039365	PIK3C2B	G=0.242	-0.147	$4x10^{-6}$	-0.176	$9x10^{-11}$										
rs4951407	MDM4	G=0.702											-0.246	$6x10^{-5}$	-0.115	$5x10^{-5}$
	PIK3C2B	G=0.702	-0.115	$8x10^{-6}$	-0.125	$2x10^{-7}$										
rs1380576	MDM4	C=0.723											-0.264	$2x10^{-5}$	-0.12	$2x10^{-5}$
	PIK3C2B	C=0.723	-0.108	$4x10^{-5}$	-0.11	$5x10^{-6}$										
rs4951393	MDM4	A=0.711											-0.271	$9x10^{-6}$	-0.135	$2x10^{-6}$
	PIK3C2B	A=0.711	-0.116	$8x10^{-6}$	-0.112	$3x10^{-6}$										
rs10494852	MDM4	T=0.725											-0.251	$7x10^{-5}$	-0.111	$1x10^{-4}$
	PIK3C2B	T=0.725	-0.103	$1x10^{-4}$	-0.117	$1x10^{-6}$										
rs10900594	MDM4	C=0.727													-0.118	$2x10^{-5}$
	PIK3C2B	C=0.727	-0.108	$4x10^{-5}$	-0.109	$6x10^{-6}$										
rs12039454	MDM4	C=0.713											-0.271	$9x10^{-6}$	-0.132	$3x10^{-6}$
	PIK3C2B	C=0.713	-0.111	$2x10^{-5}$	-0.111	$3x10^{-6}$										
rs16853958	MDM4	C=0.728													-0.118	$2x10^{-5}$
	PIK3C2B	C=0.728	-0.107	$5x10^{-5}$	-0.115	$2x10^{-6}$										
rs2369244	MDM4	C=0.728											-0.271	$9x10^{-6}$	-0.132	$3x10^{-6}$
	PIK3C2B	C=0.728	-0.111	$2x10^{-5}$	-0.113	$3x10^{-6}$										
rs4252725	MDM4	C=0.728											-0.249	$5x10^{-5}$	-0.116	$3x10^{-5}$
	PIK3C2B	G=0.728	-0.104	$7x10^{-5}$	-0.111	$5x10^{-6}$										
rs4951382	RP11-430C7.5	C=0.754													0.158	$2x10^{-5}$
	PIK3C2B	C=0.754	-0.112	$3x10^{-5}$	-0.131	$8x10^{-8}$										
rs7556371	RP11-430C7.5	A=0.749													0.152	$4x10^{-5}$
	PIK3C2B	A=0.749	-0.103	$9x10^{-5}$	-0.123	$4x10^{-7}$										

Abbreviations: SNP, single nucleotide polymorphism; LD, linkage disequilibrium. [a] SNPs in high LD (D'>0.95) with SNPs tested in GEM participants. [b] Effector genes and related data retrieved from the GTEx (Genotype-Tissue Expression) project's portal (v8 data retrieved on 10/15/2021) for not sun exposed skin tissues (suprapubic area, n=605), sun exposed skin (tissues from lower leg area,

n= 517), brain tissues from spinal cord (cervical c1, n=126); hippocampus (n=165); substantia nigra (n= 114), and lung (n= 515). No significant results were obtained for liver and small intestine. No data were available for bone.

Table S6. Interaction between MDM2 and *MDM4* SNPs on risk for developing multiple melanoma, and risk of melanoma-specific death.

*MDM2*MDM4* **SNPs**	**N**	**Exponentiated beta coeficient**	**95% CI**	$P_{Interaction}$
Interaction between MDM2 and MDM4 SNPs on risk [a]				
rs4245739 * rs117039649	3553	0.86	0.54, 1.36	0.50
rs4245739 * rs2279744	3397	1.08	0.90, 1.29	0.40
rs1563828 * rs117039649	3564	0.82	0.52, 1.29	0.40
rs1563828 * rs2279744	3399	1	0.84, 1.18	0.97
Interaction between MDM2 and MDM4 SNPs on survival [b]				
rs4245739 * rs117039649	3519	1.86	0.81, 4.24	0.14
rs4245739 * rs2279744	3363	0.92	0.67, 1.26	0.6
rs1563828 * rs117039649	3530	1.53	0.67, 3.48	0.3
rs1563828 * rs2279744	3365	0.92	0.68, 1.24	0.6

Abbreviations: N, number; CI, Confidence Interval; $P_{interaction}$, p-value for interaction term. [a] Adjusted for age, sex, age*sex interaction, and centre. [b] Adjusted for age, sex, centre, status_orig, and time-dependent covariate.

Table S7. Minimally adjusted hazard ratios for *MDM2* and *MDM4* diplotypes and melanoma-specific death by sex.

	**All** (n=3519)				**Females** (n=1547)				**Males** (n=1972)			
**Diplotypes**	**Frequency**	**HR**[a]	**95% CI**[a]	P_{global}[a]	**Fre-quency**	**HR**[c]	**95% CI**[c]	P_{global}[c]	**Fre-quency**	**HR**[c]	**95% CI**[c]	P_{global}[c]
MDM2 **rs117039649** (SNP285) - ***MDM4*** **rs4245739**												
GG-AA	1713	1.00	(ref)	0.33	763	1.00	(ref)	0.85	950	1.00	(ref)	0.15
GG-AC	1,346	0.84	0.64, 1.10		583	1.22	0.74, 2.02		763	0.72	0.52, 1.00	
GG-CC	219	0.71	0.39, 1.28		93	0.83	0.25, 2.73		126	0.66	0.33, 1.31	
GC/CC-AA	130	0.45	0.18, 1.10		55	0.82	0.20, 3.45		75	0.34	0.11, 1.09	
GC/CC-AC	93	0.97	0.45, 2.08		46	1.51	0.46, 4.95		47	0.80	0.29, 2.19	
GC/CC-CC	18	0.77	0.11, 5.54		7	<0.01	0.00, >999		11	0.97	0.13, 6.95	
$P_{interaction}$=0.14					$P_{interaction}$=0.95				$P_{interaction}$=0.09			

Abbreviations: N, number; HR, Hazard Ratio; CI, Confidence Interval; P_{global}, global p-value; $P_{interaction}$, p-value for interaction term; ref, reference group. HR, CI, and global p-values obtained from a Cox proportional hazards model adjusted for age at diagnosis, sex, study center, single or multiple primary melanoma status, plus a time-dependent variable for cases with single primary melanoma who developed a subsequent melanoma during follow-up. The referent group corresponds to the most common combined genotypes.
